# The effect on fall rate of blood glucose testing at the time of falls in elderly diabetics

**DOI:** 10.1186/1758-5996-6-65

**Published:** 2014-05-29

**Authors:** Eugene Waclawski, Nicola Cherry, Adrian Wagg

**Affiliations:** 1 Division of Preventive Medicine, Faculty of Medicine and Dentistry, University of Alberta, 8303-112 St NW, Edmonton, AB T6G 2 T4, Canada; 2 Department of Geriatric Medicine, University of Alberta, Edmonton, AB, Canada

**Keywords:** Diabetes, Falls, Blood glucose, Supportive/assisted living

## Abstract

**Objective:**

To determine the pattern of blood sugar and HbA1c testing among supportive living residents with diabetes and whether, in those with diabetes, blood glucose measurement was done at the time of a fall.

**Research design and methods:**

The management of diabetes in relation to falls in the supportive living sector is unknown. A cross-sectional questionnaire study in Edmonton Alberta, Canada of Designated Supportive Living (DSL) homes have places funded by Alberta Health Services and other homes (SL) that have no funded places. A questionnaire was distributed to Directors of Care/managers of supportive living homes, with telephone interview follow-up if required.

**Results:**

Sixty responses from 61 of the 71 homes (86%) provided information. 21 were DSL and 39 were SL homes. DSL homes were significantly more likely than SL ones to report that residents with diabetes had blood glucose measurements as part of regular care, to be aware that glycosylated haemoglobin was measured, and to say that blood glucose was measured at the time of a fall. Regression analysis identified that facilities with a policy to measure blood glucose at the time of a fall had a lower rate of falls in residents with diabetes than facilities without such a policy (p < 0.05). No effect of this policy was seen in residents without diabetes.

**Conclusion:**

Residents with diabetes were less likely to fall in homes that indicated that they had a policy to measure blood glucose at the time of a fall.

## Background

Globally, the number of people with diabetes is estimated to have increased from 153 million in 1980 to 347 million in 2008 [1]. In the United States 18.9% of the age group ≥65 years has diabetes [2]. In England in 2010 the prevalence of diabetes in those aged 65–74 was 15.7% in men and 10.4% in women and in those aged ≥75 years 13.5% and 10.6% in men and women respectively [3]. In Canada the prevalence of diabetes increased from 4.2% in 2003 to 5.1% in 2011 [4]. In 1998 the prevalence of diabetes in the elderly in Canada was estimated at 12.0% in the community, 17.5% in institutions and 12.4% overall [5].

Diabetes has been identified as one of the many factors significantly contributing to an increased likelihood of falling in older people along with cognitive impairment, depression, history of stroke, urinary incontinence, rheumatic disease, dizziness and vertigo, hypotension, fear of falling, Parkinson’s disease, comorbidities and pain [6].

Falls in people with diabetes are common; during any 12 month period, around 35% of residents in supportive living facilities fall with the proportions more than doubled (78%) for residents with diabetes mellitus compared to those without (30%) [7]. Both those with insulin-treated diabetes and those taking oral medication have a greater risk of falls than those without diabetes [8]. Data also suggest that falls suffered by those with diabetes are more likely to be injurious: in an average 7 years of total follow-up diabetic women had an increased risk of 20% of suffering a fracture having adjusted for multiple risk factors including the frequency of falls [9].

Management of falls in older people has been driven by the application of multifactorial evidence based guidelines, such as those issued jointly by the British and American Geriatrics Societies [10]; notably, the guideline does not advise any specific provision for those with a diagnosis of diabetes. The National Institute for Clinical and Healthcare Excellence for England and Wales mentions “health problems that may increase their risk of falling” as one group in which falls prevention should be adopted in UK guidance on falls but no specific mention of diabetes occurs [11].

Many reasons have been postulated for an observed greater risk of falling among those with diabetes [12], including: tight glycaemic control, as measured by HbA1c (7% or below) [13], which may indicate a higher rate of hypoglycaemic episodes [14]; impaired mobility, [15]; and an increase in the prevalence of associated co-morbid conditions, both specific to diabetes (poor vision, peripheral neuropathy) [13] and from risk factors shared with other elderly people [6].

Treatment of type 2 diabetes usually requires dietary modification with the addition of oral agents to achieve good glucose control. Insulin is added or substituted for oral agents if required to gain better control. Until recently oral agents were limited to metformin and sulphonylureas, the latter carrying a risk of hypoglycaemia. Recently new treatments for type 2 diabetes have been introduced, some of which (meglitinides) also carry a risk of hypoglycaemia [16]. As a result of concerns that hypoglycaemia contributes to the risk of falls, guidelines on management of diabetes in settings of high risk such as long-term care suggest relaxing the control of diabetes (HbA1c > 8%) [17].

The study reported here was designed to address a gap in knowledge about the effects of policies on the monitoring of diabetic control on the rate of falls in residents of supportive living homes. It aimed to determine: the prevalence of diabetes in the supportive living home population in Edmonton: the pattern (policies and practice) of blood sugar and HbA1c measurement among the residents with diabetes: and, whether, in residents with diabetes, blood glucose was measured at the time of a fall.

## Methods

In Edmonton and surrounding areas, the site of the study, all 79 supportive living homes known to the Seniors Association of Greater Edmonton [18] formed the group of homes included in this study. The term assisted living is also used to describe such homes. Supportive living provides room and board for seniors who are functionally independent or functionally independent with the assistance of community based services. The latter type of home has spaces designated for individuals who require community services and have funding provided by the province (through Alberta Health Services). In such Designated Supportive Living (DSL) homes all health and personal care services included in the resident’s personal care plan are paid for by Alberta Health Services [18]. Other supportive living homes are signified by SL in this report.

The manager or Director of Care for the facility or the Alberta Health Services care nurse of each supportive living home in the Edmonton area was sent a letter inviting them to participate in a short survey. The letter indicated the range of survey questions to allow the care home to collate the information. An information sheet and a copy of the questionnaire were included with the letter.

Unless a completed questionnaire had been mailed back, a research interviewer from the Division of Preventive Medicine phoned each facility to administer the questionnaire within 4 weeks of the letter being sent. Up to 3 call-back attempts were made. Rates of falls were calculated, for diabetic and non-diabetic residents separately, as the total number of falls/number of residents/facility/yr: multiple falls by a single resident could not be distinguished. Confidence intervals (95% CI) were calculated for rates. Frequencies of categorical factors were compared using chi-square or Fisher’s exact test with p < 0.05 indicating a significant difference. Linear regression analysis modelled the effect of diabetes monitoring strategies and polices on the rate of falls in residents with and without diabetes.

This study was approved by the University of Alberta Health Ethics Board.

## Results and discussion

**Figure 1:**
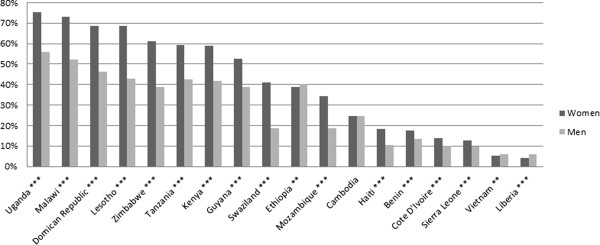
Flow diagram of results from supportive living sites (DAL – supportive living sites with funded places from Alberta health Services, SL – other supportive living sites).

Of the 79 homes invited to participate, 1 was under construction and 7 contained no seniors in receipt of supportive living. The revised study group comprised 71 eligible homes of which 7 refused to participate (5 affected by industrial action) and 3 failed to reply to repeated requests for information. Sixty one of the 71 homes provided information for this study (86% response rate). One response was provided for 2 associated facilities resulting in 60 responses for 61 facilities. Twenty one sites were DSL and 39 were SL homes.In all, 43 sites provided at least some information about the number of residents, residents with diabetes and numbers of falls. Of these, 28 sites (47% of responses) had information on resident numbers and falls including specific data on the number of falls in residents with and without diabetes and are designated below as having ‘complete’ data (Figure 1).

### Prevalence of diabetes in supportive living homes

Fifty seven sites knew the number of residents with diabetes on site. The overall prevalence in the 57 homes was 16.3% (831/5105). Among the 28 sites with complete data there were 423 residents (15.6%) with diabetes among 2713 residents. The mean prevalence of diabetes per home was 16.7% (95% CI: 8.0-25.4) for DSL homes and 18.7% (14.1-23.3) for SL homes.

### Pattern (policies and practice) of blood glucose and HbA1c measurement

Routine blood glucose measurement of people with diabetes was undertaken at 47 sites, in 20 of 21 (95.1%) DSL sites and 27 of 39 (69.2%) SL sites (Fisher’s exact test, p < 0.05). Most (31/47) responses indicated frequency of measurement was individualised to each patient. Ten suggested a standard measurement policy for all people with diabetes and 6 sites were unable to report on measurement frequency. HbA1c measurements were routinely performed at 13 (22%) sites. 8 were DSL sites (38%) and 5 were SL (13%) (p < 0.05, Fisher’s exact test).

### Blood glucose measurement at the time of a fall

A facility policy to measure blood glucose at the time of a fall existed at 22 sites. At the 21 DSL sites, 13 had this policy (62%) while at the 27 SL sites the rate was lower with only 9 sites having a facility policy (23%) (p < 0.01). All blood glucose measurements at the time of a fall were performed by supportive living home staff.

### Falls at AHS designated supportive living (DSL) and SL sites

In the 28 sites with complete data the annual fall rate per resident was very similar in residents with diabetes and for those without diabetes (Table 1). In DSL homes the rate in residents with diabetes was somewhat greater (1.27) than in SL homes (0.93) but these were again very similar to the rate for those without diabetes in homes of the same type (Table 1).

**Table 1:** Fall rate/resident/home/year in Supportive Living Homes with complete data on residents and falls (n = 28)

	**DSL homes (n = 10)**		**SL homes (n = 18)**		**All homes (n = 28)**	
	**Rate of falls**	**95% Cl**	**Rate of falls**	**95% CI**	**Rate of falls**	**95% CI**
Residents with diabetes	1.27	0.46-2.09	0.93	0.39-1.47	1.05	0.63-1.48
Residents without diabetes	1.37	0.61-2.14	1.02	0.58-1.46	1.15	0.78-1.52

DSL – Designated Supportive Living Homes; SL Other Supportive Living Homes.

Rate of falls – Rate of falls /resident/home/year.

### Modelling management and rate of falls

Linear regression modelling was carried out for residents with diabetes and without diabetes separately. Factors considered as possible determinants of falls included the policy or practice of measuring blood glucose and glycosylated haemoglobin routinely and a policy to measure blood glucose at the time of a fall. The type of facility (DSL and SL) was also included. The only factor shown to be significantly associated with a reduced rate of falls was a facility policy to measure blood glucose at the time of a fall. This was significantly related to a lower risk of falls in residents with diabetes but not in those without (Table 2). The type of facility was not significant in this model.

**Table 2:** Relation of annual fall rate to factors specific to the care home (linear regression)

**Factor**	**Standardized coefficients**	**95% confidence interval**	**Significance**
**A) Residents with diabetes**			
Blood glucose tests routinely performed	0.391	-0.115 to 1.920	0.080
Facility policy to test at time of a fall	-0.410	-1.812 to -0.310	0.043
HbA1c tests performed	-0.310	-1.690 to 0.259	0.142
Designated supportive living home	0.193	-0.457 to 1.325	0.324
**B) Residents without diabetes**			
Blood glucose tests routinely performed	-0.232	-1.463 to 0.534	0.346
Facility policy to test at time of a fall	0.072	-0.734 to 1.014	0.743
HbA1c tests performed	-0.045	-1.046 to 0.867	0.848
Designated supportive living home	-0.101	-1.072 to 0.676	0.644

## Discussion

In this survey, Designated Supportive Living homes had a similar prevalence of residents with diabetes compared to other supportive living homes (16.7% compared to 18.7%). The rates recorded are close to the institutional rate (17.5%) previously noted in Canada in 1998 [5] even though supportive living residents are considered functionally independent. This may reflect the rising prevalence of diabetes in older Canadians: in 2008 the number of Canadians ≥ 65 years of age with diabetes was 679,436 and by 2012 had increased to 894,226 (a rise of 32%) [19].

The policy and practice of management of diabetes in supportive living homes in Edmonton and area differs between those supportive living facilities with contracts for care with statutory health services (DSL), and those without (SL). DSL homes were significantly more likely than SL homes to report that residents with diabetes had blood glucose measurements performed as part of regular care and were also more likely to be aware that glycosylated haemoglobin was measured. In addition, the respondents from DSL homes were more likely to say that blood glucose was measured at the time of a fall.

The overall rates of falls for residents with diabetes were not found to be greater than those for non-diabetic residents in this study of supportive living homes. Others have reported higher rates for diabetics [7,20] and it is unclear why this was not found here. In the present study residents with diabetes were less likely to fall in homes that indicated that they measured blood glucose at the time of a fall. It may be questioned whether such a policy was reflective of an overall environment that was protective from falling. However, were this the case, residents without diabetes would also be expected to show a lower risk. They did not, suggesting that a policy of glucose measurement at the time of a fall had a protective effect that was specific to residents with diabetes

The results of the present study cannot determine the appropriate content of individual care plans for residents with diabetes but it appears that benefit, shown in fewer falls, is associated with glucose measurement at the time of a fall. We assume that, where control appears to be poor, this alerts the responsible primary care physician to review and adjust diabetic medication, thus reducing the risk of further falls in that patient. In addition to the clinical benefit of such a policy, systematic documentation of such fall-associated blood glucose results would, over time, allow investigation of the association (if any) between hypo- or hyper- glycaemia and falls in frail elderly diabetics.

This study has limitations. There was a high response rate from the homes surveyed (86%). but the information provided was complete for only 28 of the 60 responses (47%). Further, the survey did not collect information on adverse effect of falls such as injury, admission, or mortality rates and it was not possible to comment on the contribution to the overall rate of repeated falling in individual residents. The study used measurements at the level of the care home. No individual health measures were obtained. The collection of covariates will require further study ideally among residents in homes with and without management policies for diabetes in our location and by others.

The survey has identified some challenges in data provision from the supportive living sector but suggests that opportunities exist, with suitable planning and cooperation of supportive living homes, to investigate further how policy interventions might help to contain falls in this population and whether glucose measurement at the time of a fall reduces risk by triggering more appropriate glucose control.

## Conclusions

Residents with diabetes were less likely to fall in homes that indicated that they had a policy to measure blood glucose at the time of a fall.

We assume that, where control appears to be poor, this alerts the responsible primary care physician to review and adjust diabetic medication, thus reducing the risk of further falls in that patient.

## Competing interests

The authors declare that they have no competing interests.

## Author contributions

EW, NC and AW devised this study. EW managed data collection and drafted paper. EW and NC undertook statistical analysis. EW, NC and AW commented and edited the document and agreed final version.
